# Realistic Aspects of Cardiac Ultrasound in Rats: Practical Tips for Improved Examination

**DOI:** 10.3390/jimaging10090219

**Published:** 2024-09-06

**Authors:** Jessica Silva, Tiago Azevedo, Mário Ginja, Paula A. Oliveira, José Alberto Duarte, Ana I. Faustino-Rocha

**Affiliations:** 1 Centre for the Research and Technology of Agro-Environmental and Biological Sciences (CITAB), Institute for Innovation, Capacity Building and Sustainability of Agri-Food Production (Inov4Agro), University of Trás-os-Montes and Alto Douro (UTAD), 5000-801 Vila Real, Portugal; silva_jessy@hotmail.com (J.S.); tiagoazevedo@utad.pt (T.A.); mginja@utad.pt (M.G.); pamo@utad.pt (P.A.O.); 2 Animal and Veterinary Research Centre (CECAV), Associate Laboratory for Animal and Veterinary Sciences (AL4AnimalS), University of Trás-os-Montes and Alto Douro (UTAD), 5000-801 Vila Real, Portugal; 3 Centro de Investigação de Montanha (CIMO), Laboratório Associado para a Sustentabilidade e Tecnologia em Regiões de Montanha (SusTEC), Instituto Politécnico de Bragança, Campus de Santa Apolónia, 5300-253 Bragança, Portugal; 4 Department of Veterinary Sciences, University of Trás-os-Montes and Alto Douro (UTAD), 5000-801 Vila Real, Portugal; 5 Associate Laboratory i4HB, Institute for Health and Bioeconomy, University Institute of Health Sciences (IUCS), Advanced Polytechnic and University Cooperative (CESPU), 4585-116 Gandra, Portugal; jose.duarte@iucs.cespu.pt; 6 UCIBIO—Applied Molecular Biosciences Unit, Translational Toxicology Research Laboratory (1H-TOXRUN), University Institute of Health Sciences (IUCS), Advanced Polytechnic and University Cooperative (CESPU), 4585-116 Gandra, Portugal; 7 Department of Zootechnics, School of Sciences and Technology, University of Évora, 7004-516 Évora, Portugal; 8 Comprehensive Health Research Center (CHRC), University of Évora, 7004-516 Évora, Portugal

**Keywords:** echocardiography, murine models, heart function

## Abstract

Echocardiography is a reliable and non-invasive method for assessing cardiac structure and function in both clinical and experimental settings, offering valuable insights into disease progression and treatment efficacy. The successful application of echocardiography in murine models of disease has enabled the evaluation of disease severity, drug testing, and continuous monitoring of cardiac function in these animals. However, there is insufficient standardization of echocardiographic measurements for smaller animals. This article aims to address this gap by providing a guide and practical tips for the appropriate acquisition and analysis of echocardiographic parameters in adult rats, which may also be applicable in other small rodents used for scientific purposes, like mice. With advancements in technology, such as ultrahigh-frequency ultrasonic transducers, echocardiography has become a highly sophisticated imaging modality, offering high temporal and spatial resolution imaging, thereby allowing for real-time monitoring of cardiac function throughout the lifespan of small animals. Moreover, it allows the assessment of cardiac complications associated with aging, cancer, diabetes, and obesity, as well as the monitoring of cardiotoxicity induced by therapeutic interventions in preclinical models, providing important information for translational research. Finally, this paper discusses the future directions of cardiac preclinical ultrasound, highlighting the need for continued standardization to advance research and improve clinical outcomes to facilitate early disease detection and the translation of findings into clinical practice.

## 1. Introduction

Echocardiography is a medical imaging technique that uses sound waves to generate images of the heart [1]. Since its inception, it has evolved significantly in terms of technology and applications. The first echocardiogram in humans was successfully recorded in 1953 by Inge Edler and Carl Hellmuth Hertz, marking the birth of modern echocardiography [2]. This technique is rooted in ultrasound technology, which was developed in the early 20th century [3]. The first form of echocardiography was motion mode (M-mode), which produced one-dimensional images of the heart [3]. These images allowed for measurements of heart chamber sizes and valvular motion. In the early 1960s, two-dimensional (2D) echocardiography greatly improved imaging by enabling cross-sectional visualization of the heart’s structures [4]. In 1973, M-mode was used for the first time to determine left ventricular regional wall motion abnormalities [4,5]. Subsequent innovations, such as color and spectral Doppler techniques [6] and transesophageal echocardiography [4], expanded the clinical applications in the 1970s. The addition of three-dimensional (3D) echocardiography has revolutionized cardiac imaging, providing real-time, dynamic, and volumetric images for more accurate assessments of cardiac function and anatomy [5,6]. Echocardiography has progressed from a one-dimensional, experimental technique to a sophisticated imaging modality that is pivotal in diagnosing and monitoring various cardiovascular conditions [6,7]. Its non-invasive nature, real-time imaging, and ongoing technological advancements have made it an indispensable tool in cardiology [5].

Rodents, particularly mice and rats, are valuable models for cardiovascular research due to their well-characterized genome, uniform study populations, reproducible pathological phenotypes, and the ease of creating genetically modified models [8]. Housing rodents is also cost-effective compared to larger animals, making them accessible for a wide range of research budgets [8,9]. Surgical techniques that induce myocardial overload, infarction, and dysfunction in mice and rats facilitate the reliable identification and assessment of key physiological, molecular, and biochemical mechanisms underlying cardiovascular diseases [10]. Additionally, assessing the effects of cardiac therapies on non-cardiac models can reveal potential side effects [11]. Non-invasive imaging techniques, such as echocardiography, have facilitated comprehensive cardiovascular evaluations in rodents. This has contributed to the translational development of novel diagnostic methods and therapeutic strategies aimed at predicting and preventing complications of cardiovascular diseases in humans [9]. Advancements in this technology have successfully addressed key challenges encountered in rodent cardiac imaging. These challenges include the small size of the animal and the high heart rates observed. These obstacles have been overcome through the development of high-frequency transducers with improved signal processing and superior imaging frame rates.

Ultrasonography has proven advantageous in several studies, including those related to cardiovascular diseases, research techniques and physiology, phenotyping transgenic mice, and drug research and development [12,13,14,15,16]. Echocardiography can also be used to evaluate the heart’s response to stress, aiding in the assessment of interventions, such as exercise or pharmacological treatments on cardiac function in real-time preclinical research, including treatments in fields such as oncology and neurology [17,18].

Although this article primarily provides a guide and practical tips for the appropriate acquisition and analysis of echocardiographic parameters in adult rats, the information provided can also be applied to other rodent models, including mice. It includes considerations for animal preparation, such as anesthesia and physiological monitoring. Additionally, it provides a practical and detailed guide for using ultrasound to evaluate cardiac structure and function, describing several cardiovascular parameters applicable in preclinical models.

## 2. Principles of Echocardiography

Transthoracic imaging involves the use of high-frequency sound beams that penetrate the thoracic cavity and reflect back to the ultrasound transducer when they reach an interface between tissues of different acoustic impedance, such as the myocardium, valves, and blood. This reverberated signal is then processed by the software, producing a real-time image of the heart. In adult rats, the myocardium reflects more ultrasound than the blood.

Echocardiography, like other medical imaging techniques, has advantages and limitations, as shown in (Table 1). It is non-invasive and provides real-time images of the heart’s structure and function, allowing for immediate assessment and diagnosis during the examination [12,14,15,16,19,20,21,22,23,24,25]. Additionally, the portable equipment enhances usability in terms of both space and time, allowing for examinations on both awake and anesthetized animals with ease [12,13,14,15,16,19]. A wide variety of cardiac measurements can be taken [19,25,26], and the resulting images are highly reproducible, making it suitable for monitoring changes in cardiac function over time or comparing results before and after treatment [14,16,19,22,27,28]. Echocardiography is often considered a more cost-effective alternative to other cardiac imaging techniques, such as cardiac magnetic resonance imaging (MRI) or computed tomography (CT) [12,15,19,21,26,27].

Although echocardiography provides valuable insights into cardiac structure and function, it also has certain limitations. Firstly, the procedure can be time-consuming, sometimes exceeding 20 min, even when no abnormalities are detected [24]. This extended duration is due to multiple measurement parameters, which add complexity and subjectivity to the process [24]. Moreover, echocardiography is considered an operator-dependent technique, which means that the quality of images and measurements can vary depending on the operator’s skills and experience, potentially leading to inconsistencies in the results [12,24]. Therefore, this technique requires the expertise of a medically trained specialist to ensure accuracy and reliability.

Despite its limitations, echocardiography remains a versatile tool for evaluating cardiac function and diagnosing various cardiac diseases, including valvular heart disease [8,29,30], cardiomyopathies [8,9,30,31], congenital heart disease [8,29,31], ischemic heart disease [8,9,30,32], cardiac tumors [30], pulmonary hypertension [8,30,33,34], and cardiac transplants [30]. Its significance lies in providing valuable information for treatment planning, monitoring disease progression, and assessing intervention effectiveness. This establishes it as a fundamental component of modern cardiology practice [14,19,22].

## 3. Practical Aspects of Echocardiography in Rodent Research

When recording ultrasound images, it is crucial to maintain the animals’ calmness, as even minor movements can interfere with signal acquisition, resulting in inaccurate results. Therefore, immobilization is necessary. There are several factors to consider when preparing animals for anesthesia, including the drugs’ selection, induction, maintenance, and recovery of the anesthesia, as well as post-anesthetic care [35]. The success and reliability of echocardiography studies in rodents depend on several factors, including the administration of anesthesia, the choice of drugs and administration route, and the positioning of the animal. It is important to note that all procedures carried out on animals outside of their normal routine can cause stress and lead to harmful effects on studies. Therefore, compliance with the 3Rs should be ensured.

### 3.1. Anesthesia

In rodent research, echocardiography can be performed in two ways: on awake animals or on those under anesthesia. When performed on awake animals, acclimatization to the imaging environment is required in order to get the animals familiarized with the procedures, especially when conducting serial measurements on the same individual, to ensure a stress-free environment for accurate and consistent results [12,19]. When conducted under anesthesia, it is essential to monitor the animals carefully to prevent suffering and maintain a heart rate (HR) between 400 and 650 beats per minute [12], ensuring the ethical and effective use of echocardiography in rodent studies.

Anesthesia serves the critical role of immobilizing animals and reducing stress and anxiety. It can be administered through continuous inhalation of a gas or intraperitoneal injection. The choice of anesthetic significantly affects cardiac function during rodent echocardiography [36]. It is essential to ensure accurate dosage based on the animal’s weight to guarantee a safe procedure and facilitate a smooth recovery. The anesthetics commonly used for mouse echocardiography are isoflurane, avertin, ketamine, and xylazine [36,37]. Isoflurane is a commonly used anesthetic for both initiating and maintaining anesthesia [19]. Animals are typically exposed to this anesthetic in an induction chamber at 20 °C [38]. Our research team has successfully used a combination of ketamine (Imalgene^®^ 1000, Merial S.A.S., Lyon, France) and xylazine (Rompun^®^ 2%, Bayer S.A., Kiel, Germany) at a dose of 75 mg/kg and 10 mg/kg, respectively, via the intraperitoneal route to anesthetize rats for echocardiographic examination. When selecting an anesthetic, it is crucial to consider the study’s specific requirements and its potential impact on cardiac function, including blood pressure, HR, cardiac function, and recovery time [12]. Additionally, it is important to consider the effects on cerebral metabolism, as most anesthetic agents suppress cerebral metabolism. To ensure the animal’s wellbeing during the procedure, it is critical to perform regular physiological monitoring, ideally every 15 min [12,35].

### 3.2. Animal Positioning

Accurate positioning of animals is essential for obtaining optimal-quality ultrasound images. This task should be undertaken by researchers with relevant experience. Furthermore, it is imperative to maintain the animals’ body temperature at 37 ± 0.5 °C, which can be achieved by placing the animal on a heating pad in a supine position [16]. To prevent the eye region from drying out, it is recommended to apply eye lubricant to each eye. Before beginning the imaging process, it is important to prepare the area by removing hair with a depilatory cream or carefully shaving with an appropriately sized clipper [39]. Then, apply pre-warmed ultrasound gel to the chest area at the imaging location.

To carry out echocardiography on the rat during experimental trials, after anesthesia, as indicated above, our research group places the animal in the supine position and trichotomizes the thoracic region using a machine clipper (AESCULAP^®^ GT420 Isis; Aesculap Inc., Center Valley, PA, USA). Then, the acoustic gel (Aquasonic^®^; Parker Laboratories Inc., Fairfield, NJ, USA) is applied to the region to be examined.

### 3.3. Ultrasound Equipment

Ultrasound equipment is a medical device that utilizes high-frequency sound waves to produce images of internal body structures. The equipment emits and receives tissue reflections of ultrasound waves, forming the echocardiography images [12,40]. For echocardiography imaging in adult mice, it is recommended to use transducers with a frequency between 30 and 40 MHz for body weights less than 35 g. This ensures the maintenance of a real-time imaging frame rate exceeding 30 frames per heartbeat [41]. For adult rats, frequencies between 10 and 25 MHz are considered appropriate for obtaining high-quality echocardiographic images [42]. Our research team uses a real-time scanner (Logic P6^®^; General Electric Healthcare, Milwaukee, WI, USA) with a 4–10 MHz linear probe (Model I739, General Electric Healthcare, Milwaukee, WI, USA) for echocardiography in rats. To avoid potential artifacts in echocardiography, it is important to consider several other factors related to ultrasound equipment. These factors include controlling ambient light levels in the room, ensuring heart rates remain above 350 beats per minute (bpm) for rats and 450 bpm for mice, and preventing animal hypothermia. It is crucial to highlight that the recommendations are applicable to other comparable equipment available on the market, with similar outcomes anticipated.

### 3.4. Echocardiography Modes

#### 3.4.1. B-Mode

Brightness mode (B-mode) is the simplest form of echocardiography. It generates real-time black-and-white images of the heart and other vasculatures, such as the carotid artery, pulmonary artery, and aortic arch [14]. B-mode enables the non-quantitative evaluation of cardiac physiology, cardiac chamber dimensions, and cardiac anatomical components [12], as well as the systolic function [43]. The program and equipment can gather multiple measurements from the generated images. The ultrasound probe can be linked to a motor, allowing for 3D visualization through the combination of 2D images [44].

#### 3.4.2. M-Mode

M-mode echocardiography is a technique that involves tracing B-mode scan images continuously along a single line or axis. This provides a visual representation of myocardial wall movement during systole and diastole [12]. It evaluates the systolic function and left ventricular (LV) size in parasternal long-axis (PLAX) and parasternal short-axis (PSAX) views. The M-mode tracings are optimal at the papillary muscle level. A circular LV shape in the PSAX view indicates proper imaging, while an oval appearance indicates oblique imaging.

#### 3.4.3. Doppler Mode

Doppler imaging is a technique used in echocardiography to assess blood flow within the heart and blood vessels [45]. It involves measuring the change in frequency of sound waves as they reflect off moving objects, such as red blood cells. This information is then used to create images and quantify blood flow velocities [7,46]. Pulsed wave and color Doppler focus on blood cells, while tissue Doppler imaging assesses myocardial tissue movement.

#### 3.4.4. Speckle-Tracking Echocardiography

Speckle-tracking echocardiography is a non-invasive technique that enables precise assessment of cardiac function and myocardial mechanics. It offers valuable insights into cardiac physiology and pathology [47] by tracking the movement of natural acoustic markers within the myocardium, known as “speckles”, over the cardiac cycle. This method provides detailed insights into temporal resolution compared to traditional echocardiographic techniques [48]. Speckle-tracking echocardiography is particularly invaluable in small animal models like rats, where accurate measurements are vital for detecting subtle changes in cardiac function [49]. This technique has become a powerful tool in preclinical research, enabling a comprehensive assessment of cardiac mechanics. It has contributed significantly to our understanding of cardiovascular diseases and the development of new therapeutic approaches [50].

#### 3.4.5. Three-/Four-Dimensional Imaging

Three-dimensional imaging is an advanced imaging technique that provides a more comprehensive and detailed view of the heart compared to traditional 2D echocardiography. This allows clinicians to assess the heart’s anatomy, function, and blood flow dynamics, visualizing the cardiac chambers (atria and ventricles) and the heart valves [40]. The technique uses sound waves to create a real-time 3D image of the heart and its structures [15,51]. Three-dimensional echocardiography captures a volume of data, resulting in a more comprehensive and realistic representation of the heart [52]. This is particularly beneficial for assessing complex cardiac structures and abnormalities [53], such as ventricular volumes, ejection fraction, and regional wall motion abnormalities [54]. Although 3D echocardiography offers significant advantages, it also presents challenges. These include the need for specialized equipment, increased data processing requirements, and a steep learning curve for operators. Additionally, interpretation and analysis of 3D images may require expertise [53].

Four-dimensional echocardiography, also known as real-time three-dimensional echocardiography, provides clinicians and researchers with real-time dynamic visualization of cardiac structures and function. This imaging technique offers improved spatial resolution and valuable insights into complex cardiac anatomy and pathology for clinicians and researchers [55,56]. This technology allows for more precise evaluation of cardiac conditions and aids in improved surgical planning and interventions, particularly in cases of congenital heart defects, valvular diseases, and cardiomyopathies [57]. However, it requires a high-quality ultrasound machine and specialized training, making it less standardized in preclinical models due to its higher cost. At the time of writing, there are several companies offering a diverse range of premium ultrasound equipment, including probes that facilitate the acquisition of high-quality 4D images.

## 4. Data Collection and Analysis

**Figure 1:**
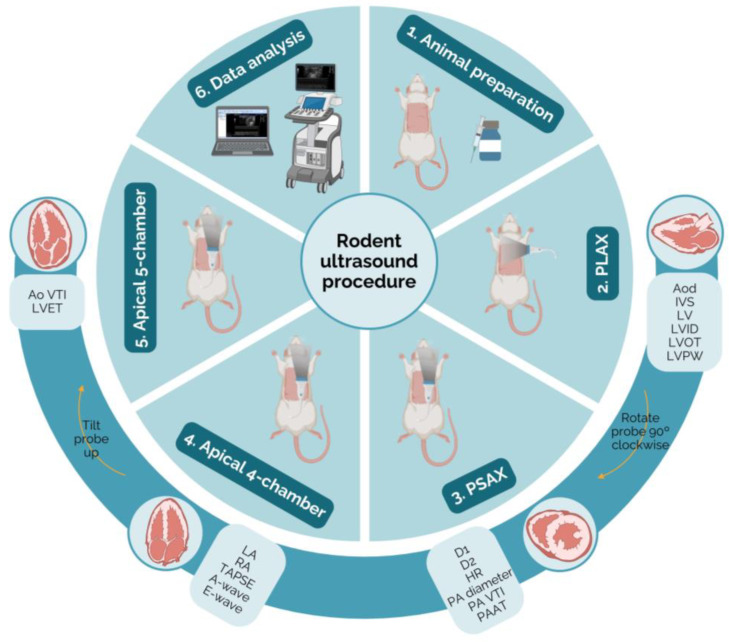
Schematic representation of the protocol for echocardiography in rodents. The probe positioning, the heart ultrasound image, and the parameters measurable in each view may be observed (created with BioRender.com, accessed on 10 April 2024). Ao VTI—aortic velocity–time integral; Aod—aorta diameter; D1—left ventricle short-axis diameter parallel; D2—left ventricle short-axis diameter perpendicular; HR—heart rate; IVS—intraventricular septum thickness; LA—left atrium; LV—left ventricle; LVET—left ventricle ejection time; LVID—left ventricle internal diameter; LVOT—left ventricular outflow tract; LVPW—left ventricle posterior wall thickness; PA diameter—pulmonary artery; PA VTI—pulmonary artery velocity–time integral; PAAT—pulmonary artery acceleration time; PLAX—parasternal long axis; PSAX—parasternal short axis; RA—right atrium; TAPSE—tricuspid annular plane systolic excursion.

Following the considerations outlined above, a comprehensive echocardiographic examination can be conducted to evaluate heart function in rats. This is crucial in experimental studies that aim to comprehend the pathophysiology of cardiac diseases and investigate the effects of innovative therapies. To perform this examination, images from four transthoracic echocardiography views should be acquired sequentially: PLAX, PSAX, apical four-chamber, and apical five-chamber. It is important to maintain a consistent order and timing across individuals to reduce variability, although the sequence may be chosen according to personal preferences and data acquisition priorities. The parameters may be measured using electronic cursors integrated into the ultrasound apparatus or exported and measured using a free MicroDicom 2023.1 viewer and software (Figure 1).

### 4.1. Measurable Parameters in Rat Echocardiography

#### 4.1.1. Parasternal Long-Axis (PLAX) View

The PLAX view offers a complete longitudinal cross-section of the heart, encompassing the ventricles, atria, and interventricular septum [12,58]. To obtain this view, the transducer should be positioned over the left third of the animal’s chest wall, aligning the notch toward the animal’s right shoulder [47,59]. The PLAX view allows the measurement of aorta diameter (Aod, mm) during diastole. This is performed by using B-mode or M-mode in the aortic root during diastole [14,16,45,47]. An elevated Aod indicates an increased risk of adverse events [39,58], which may suggest the presence of an aortic aneurysm [60]. This measurement is crucial for assessing the risk and extent of aortic dissection, particularly in conditions such as aortic stenosis or regurgitation, which are influenced by aortic valve function [61].

*How to measure Aod*

**Figure 2:**
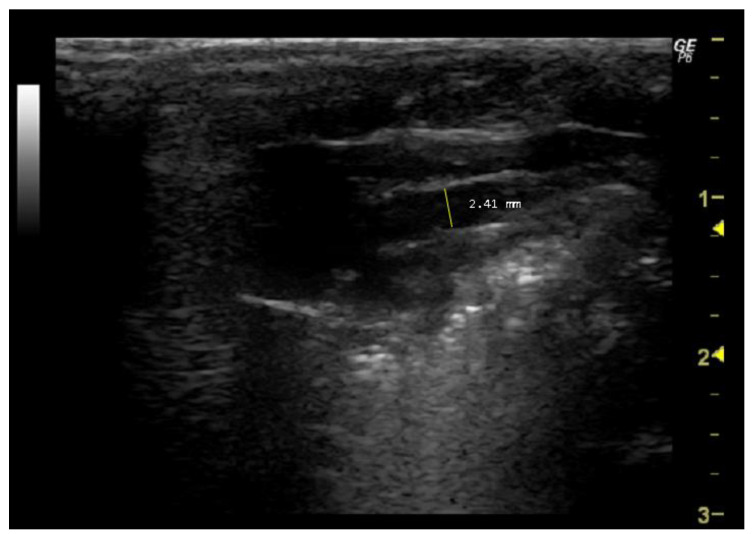
Aorta diameter (Aod, mm) measured in 26-week-old female Wistar rats using B-mode at diastole, obtained in PLAX view. The image was obtained using a real-time scanner (Logic P6^®^; General Electric Healthcare, Milwaukee, WI, USA) with a 4–10 MHz linear probe. Measurements were taken using MicroDicom 2023.1 viewer and software.

To measure this parameter, it is first necessary to identify the aorta in the echocardiographic image. Typically, the aorta is visible adjacent to the left ventricle and may appear as a tubular structure with bright echogenic walls. To ensure accurate measurement, the mitral valve (left atrioventricular valve) must be visualized in its open position during diastole. The cursor should be placed perpendicular to the long axis of the aorta, from the inner edge of the anterior wall to the inner edge of the posterior wall, using the measurement tools provided by the software (Figure 2).

Interventricular septum thickness (IVS, mm) is another important echocardiographic parameter that is measured in PLAX view using B-mode. It assesses the thickness of the muscular wall between the left and right ventricles during systole and diastole [62]. IVS in diastole (IVSd) is measured just before systole onset [19], while IVS in systole (IVSs) is measured at the peak of systole [19]. Abnormalities in the IVS may indicate conditions such as hypertrophic cardiomyopathy [63]. Assessing both the IVSs and IVSd provides dynamic insights into how the septum behaves throughout the cardiac cycle.

Regarding left ventricle parameters, the left ventricle internal diameter (LVID, mm) is a crucial parameter that provides valuable information about the size, contractility, and overall performance of the left ventricle [64]. This measurement is typically taken in the PLAX view with B-mode at systole or diastole [62] and reflects the left ventricle’s internal dimension at the end of systole and diastole. During diastole, LVID (LVIDd) reflects the left ventricle’s capacity to fill with blood in its relaxed state [65], while during systole (LVIDs) indicates the degree of contraction and ejection of blood from the left ventricle [54]. These measurements provide important information about left ventricular function. Monitoring LVID can aid in the diagnosis and monitoring of conditions like hypertrophic cardiomyopathy, as well as predict cardiovascular events such as myocardial infarction [63,66,67].

Left ventricle posterior wall thickness (LVPW, mm) is measured in the PLAX view with B-mode at systole and diastole [62]. This measurement provides valuable information about the dynamics of the left ventricle throughout the cardiac cycle [65]. At diastole, LVPW indicates maximum posterior wall thickness, which helps assess the left ventricle’s ability to fill with blood [68]. During systole, it represents the minimum posterior wall thickness, indicating the degree of contraction and the extent of blood ejection from the left ventricle [69]. Increased LVPW, especially in diastole, may indicate left ventricular hypertrophy, which is associated with conditions such as hypertension, aortic stenosis, or hypertrophic cardiomyopathy. Monitoring LVPW over time can aid in evaluating changes in cardiac structural, disease progression, and treatment effectiveness [70].

*How to measure IVS, LVID, and LVPW*

**Figure 3:**
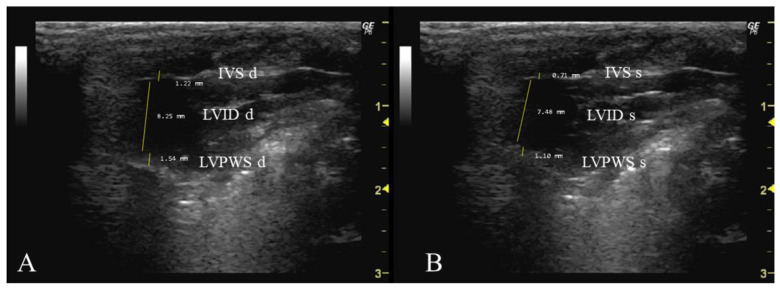
Interventricular septum thickness (IVS, mm), left ventricle internal dimension (LVID), and left ventricle posterior wall thickness (LVPW) measured in 26-week-old female Wistar rats using B-mode in diastole (**A**) and systole (**B**), obtained in PLAX view. The images were obtained using a real-time scanner (Logic P6^®^; General Electric Healthcare, Milwaukee, WI, USA) with a 4–10 MHz linear probe. Measurements were taken using MicroDicom 2023.1 viewer and software.

In order to measure these three parameters, it is necessary to select the same image as the one used for the previous parameter (Aod). This should be performed during two phases of the cardiac cycle: when the mitral valve is open (diastole) (Figure 3A) and when it is closed (systole) (Figure 3B). Firstly, the interventricular septum, which separates the LV into two chambers, and the LV cavity should be identified. To measure IVS, the cursor should be placed perpendicular to the septum, extending from the inner edge of the endocardium to the inner edge of the opposite endocardium. For the measurement of LVID, the cursor should be placed perpendicular to the long axis of the LV, extending from the inner edge of the anterior wall to the inner edge of the posterior wall. Lastly, to measure LVPW, the cursor should be placed perpendicular to the posterior wall, from the inner edge of the endocardium to the inner edge of the epicardium.

Some infrequently used parameters in PLAX include the measurement of the right ventricular outflow tract (RVOT) length at end diastole. This is located just proximal to the pulmonary valve (PV) and serves as an indicator of right ventricle (RV) size, with dilation suggesting right ventricular volume overload [71]. Another parameter, the right ventricular outflow tract velocity–time integral (RVOT VTI), gauges blood flow through the RVOT. To measure RVOT VTI, the area under the curve in a pulsed wave Doppler image of PLAX should be assessed. RVOT VTI is a proxy for RV stroke volume and can be used to determine pulmonary arterial compliance in relation to pulmonary artery systolic pressure [72]. Pulmonary valve diameter (PV diameter) assesses the length of the PV in millimeters and can be measured using PLAX or PSAX view, with PLAX typically offering better visualization. PV diameter helps diagnose pulmonary valve stenosis or dilation, and when combined with PV velocity–time integral, it allows calculation of PV stroke volume and cardiac output [71]. The left ventricular outflow tract length (LVOT) measures the left ventricular outflow tract, just proximal to the aortic valve, at end diastole in a PLAX B-mode image [71]. LVOT is valuable for mouse models of aortic stenosis as it facilitates the calculation of aortic valve area.

#### 4.1.2. Parasternal Short-Axis (PSAX) View

To obtain the PSAX, rotate the transducer 90 degrees from the PLAX view so that the transducer’s notch is directed to the animal’s left shoulder. This will provide a cross-sectional image of the heart [40,47,58,59,73]. The left ventricular end-diastolic and end-systolic dimensions, wall thickness, myocardial contractility of the left ventricle, showcasing left and right ventricular outflow tracts, great vessels, aortic and pulmonary valves, pulmonary artery branching, and the aortic arch can be assessed using it [39,40,42,59].

Heart rate (HR, bpm) is an important parameter measured through the PSAX view in M-mode [39,47]. It can be influenced by various factors, including hormones, age, autonomic innervation, fitness levels, and heart diseases [74]. The use of anesthesia during echocardiography can lower HR, making it important to keep track of this parameter. Monitoring HR during echocardiography is crucial for detecting cardiovascular system alterations and helps ensure that the anesthetic depth is appropriate [45,75]. Rodents normally have higher HR values than humans [39,40,47]. In rats, HR can range from 300 to 400 beats per minute, while in mice, they can range from 350 to 700 bpm [39,59,73].

*How to measure HR*

**Figure 4:**
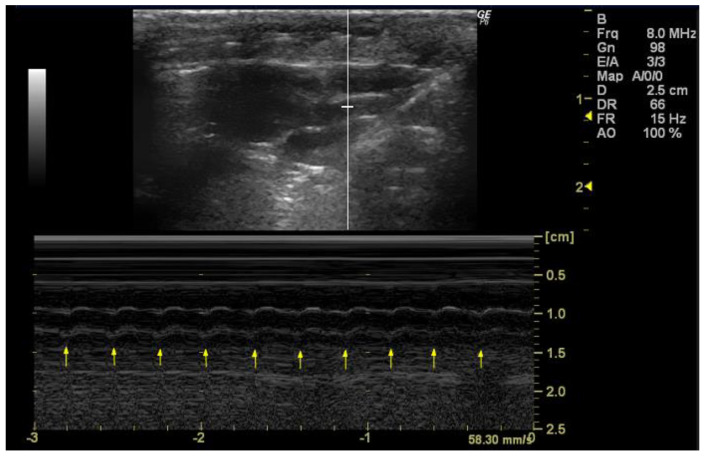
Heart rate (HR, bpm) measured in 26-week-old female Wistar rats using M-mode, obtained in PSAX view. The arrows represent the number of systoles captured in 3 seconds. The image was obtained using a real-time scanner (Logic P6^®^; General Electric Healthcare, Milwaukee, WI, USA) with a 4–10 MHz linear probe. Measurements were taken using MicroDicom 2023.1 viewer and software.

In order to accurately measure the HR, it is essential that the echocardiographic image provides a clear and detailed visualization of the heart chambers, with a particular focus on the left ventricle. Cardiac cycles should be identified by observing the repetitive movements of cardiac structures, such as the mitral valve or aortic valve, throughout the image (Figure 4). Once the image has been analyzed and captured during diastole, the number of systoles must be counted within the three-second interval corresponding to the *x*-axis/capture time displayed in the image. Following this count, the HR is calculated using the following formula:

$$HR\;\left(bpm\right)=\frac{\left(number\;of\;systoles\times 60\;s\right)}{3\;s}$$

The measurement of the left ventricle short-axis diameter, obtained in both parallel (D1, mm) and perpendicular (D2, mm) orientations to the septum, in PSAX view during B-mode echocardiography at diastole [76], is crucial for a comprehensive assessment of cardiac structure and dynamics [77]. These measurements reflect the size and geometry of the left ventricle in cross-section [78], and changes in diameter may indicate alterations in ventricular volume and shape [77].

*How to measure D1 and D2*

**Figure 5:**
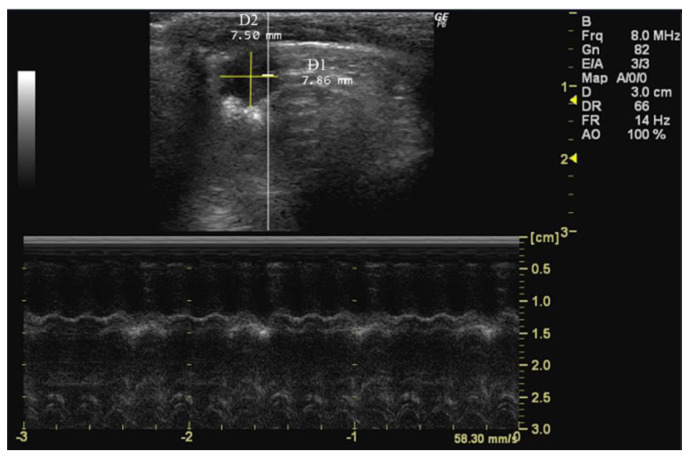
Left ventricle short-axis diameter parallel (D1) and perpendicular (D2) to the septum measured in 26-week-old female Wistar rats using B-mode at diastole, obtained in PSAX view. The image was obtained using a real-time scanner (Logic P6^®^; General Electric Healthcare, Milwaukee, WI, USA) with a 4–10 MHz linear probe. Measurements were taken using MicroDicom 2023.1 viewer and software.

To measure D1 and D2, the septum, which divides the right side from the left side of the heart, must be first located. For the D1 measurement, the cursor should be placed in a parallel orientation with the interventricular septum and perpendicular to the ventricular walls. Conversely, for the D2 measurement, the cursor should be adjusted to a perpendicular orientation relative to the interventricular septum, aligning it with the ventricular walls (Figure 5).

Pulmonary artery acceleration time (PAAT, cm/s) is a key parameter for assessing pulmonary artery hemodynamics [79] and pressure [80]. It measures the duration of initial blood flow acceleration from the pulmonary valve to the main pulmonary artery, commonly used to evaluate right ventricular afterload [79,80,81]. A shortened PAAT is associated with increased pulmonary artery pressure, which is 10–50 observed in conditions such as pulmonary hypertension [82]. PAAT is obtained in PSAX view with Pulsed Doppler mode [80].

*How to measure PAAT*

**Figure 6:**
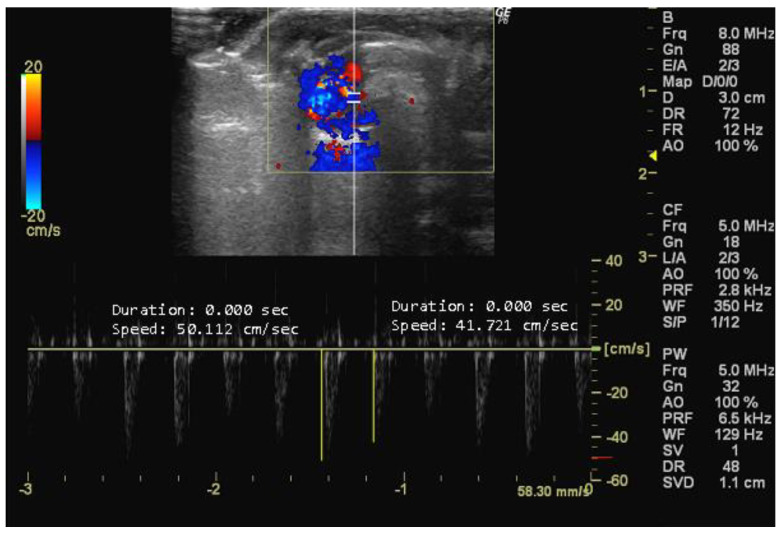
Pulmonary artery acceleration time (PAAT, cm/s) measured in 26-week-old female Wistar rats using Doppler mode, obtained in PSAX view. The image was obtained using a real-time scanner (Logic P6^®^; General Electric Healthcare, Milwaukee, WI, USA) with a 4–10 MHz linear probe. Measurements were taken using MicroDicom 2023.1 viewer and software.

To accurately measure PAAT, it is essential to identify the pulmonary artery within the echocardiographic image. Typically, the pulmonary artery is located in close proximity to the aorta, coursing anteriorly and superiorly from the right ventricle. Doppler imaging is essential for the identification of the flow velocity waveform within the pulmonary artery, which corresponds to the onset of ejection from the right ventricle. The time interval between the onset of ejection and the peak flow velocity in the pulmonary artery represents the PAAT (Figure 6).

Doppler mode also allows the evaluation of pulmonary artery diameter, which is important for heart-to-lung blood supply [83,84]. This artery, which is thicker in rats due to the presence of striated muscle fibres that are contiguous with those of the heart [83], depends on the pulmonary valve to prevent blood backflow during diastole [84]. Pulmonary artery diameter is obtained in the PSAX view with the pulsed wave Doppler mode [39,40].

*How to measure pulmonary artery diameter*

**Figure 7:**
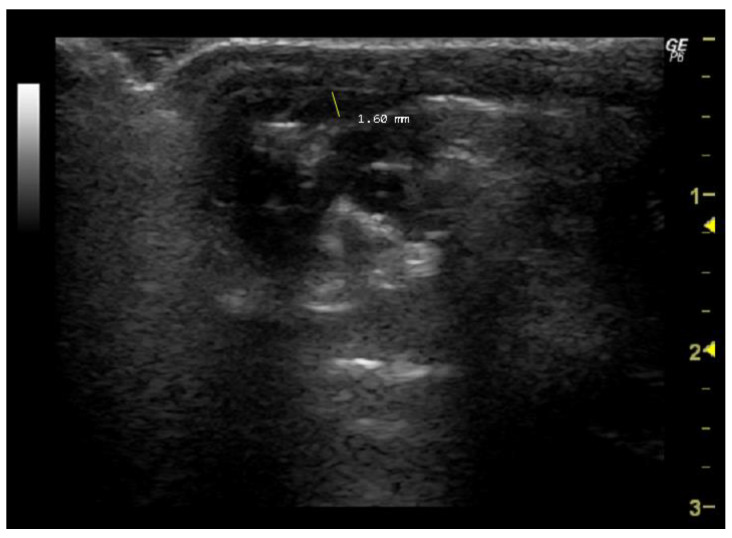
Pulmonary artery diameter (mm) measured in 26-week-old female Wistar rats using Doppler mode, obtained in PSAX view. The image was obtained using a real-time scanner (Logic P6^®^; General Electric Healthcare, Milwaukee, WI, USA) with a 4–10 MHz linear probe. Measurements were taken using MicroDicom 2023.1 viewer and software.

To measure this parameter, it is first necessary to visualize the pulmonary artery in a manner similar to that employed in the measurement of PAAT. Subsequently, the site along the pulmonary artery where the diameter appears largest and most easily measurable must be selected. This point is often in close proximity to the level of the pulmonary valve or main pulmonary artery. Then, the cursor should be placed perpendicular to the long axis at the widest point of the vessel, extending from the inner edge of the anterior wall to the inner edge of the posterior wall (Figure 7).

The pulmonary artery velocity–time integral (PA VTI, cm), obtained in PSAX view using Doppler mode [39,58,59,62,85], provides information on blood flow velocity in the pulmonary artery [39,59,62]. It assesses right ventricular systolic function and pulmonary hemodynamics [39] and offers insights into stroke volume, reflecting the amount of blood ejected into the pulmonary circulation during each cardiac cycle [86]. A reduction in PA VTI may suggest a worse prognosis, especially in patients with pulmonary hypertension [59].

#### 4.1.3. Apical View

The apical view is obtained by positioning the transducer at the fifth intercostal space in the left hemithorax [47]. This view allows for the assessment of cardiac wall motion and blood flow [45]. In this mode, the operator commonly uses B-mode to locate the structure and then switches to other modes.

##### Apical Four-Chamber View

The apical four-chamber view is useful for assessing chamber dimensions and wall motion, as it displays all four heart chambers, allowing for simultaneous visualization of both atria and ventricles [45,58]. To obtain this view, the transducer should be placed at the cardiac apex and oriented toward the animal’s right scapula. Ensure that the transducer’s notch faces the animal’s left axilla to obtain a 2D apical four-chamber view. When properly adjusted, this image displays the four chambers, both atrioventricular valves, and the interventricular and interatrial septa.

Tricuspid annular plane systolic excursion (TAPSE, cm) is an indicator of right ventricle longitudinal contractile function and reflects global cardiac function [16,58,59]. To measure TAPSE, the transducer should be positioned along the right ventricle free wall perpendicular to the apex, and the annulus displacement between end diastole and end systole during a cardiac cycle should be recorded [58,59]. TAPSE has strong reproducibility, minimal inter-operator variability, and exhibits minor variation across sex and body surface area [21,54]. Therefore, it is relevant in conditions such as pulmonary hypertension and right ventricular myocardial infarction, where reduced TAPSE indicates right ventricular dysfunction [54]. This measurement is obtained in an apical 4-chamber view using M-mode during systole [52,58,59].

*How to measure TAPSE*

**Figure 8:**
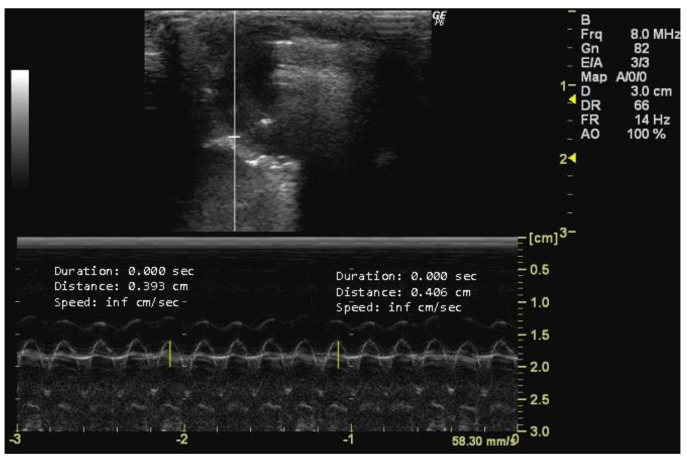
Tricuspid annular plane systolic excursion (TAPSE, cm) measured in 26-week-old female Wistar rats using M-mode at systole, obtained in apical 4-chamber view. The image was obtained using a real-time scanner (Logic P6^®^; General Electric Healthcare, Milwaukee, WI, USA) with a 4–10 MHz linear probe. Measurements were taken using MicroDicom 2023.1 viewer and software.

To measure TAPSE, it is essential to obtain an echocardiographic image that allows for the clear visualization of both the tricuspid valve and the right ventricle. In this image, the tricuspid valve should be positioned in the center, with the right ventricle adjacent to it. The M-mode imaging modality should be used, and the cursor should be placed and activated perpendicular to the septal tricuspid annulus, ensuring that it intersects the annulus and the right ventricle free wall to obtain a single-line tracing of the motion over time. The distance from the baseline (end-diastolic position) to the peak systolic position of the right ventricle free wall motion represents TAPSE (Figure 8).

The peak early diastolic transmitral flow, known as E-wave, represents the velocity of blood flow velocity when the left ventricle relaxes during early diastole [43]. This measurement provides important information about left ventricular diastolic function [14,45] and can detect changes in diastolic filling dynamics related to various cardiac conditions [65,87]. Conversely, the peak late diastolic transmitral flow, known as A-wave, is the peak blood flow velocity at end diastole caused by atrial contraction [43]. The A-wave contributes to the assessment of left ventricular diastolic function [26,88], following the E-wave during early diastole [16,26]. Changes in A-wave velocity indicate alterations in atrial function and conditions affecting diastolic filling dynamics [6,58]. Both measurements can be obtained in apical 4-chamber view using Doppler mode during the early phase of diastole by accurately positioning the sample volume at the mitral valve leaflets’ apex [6,89,90].

*How to measure E and A peaks*

**Figure 9:**
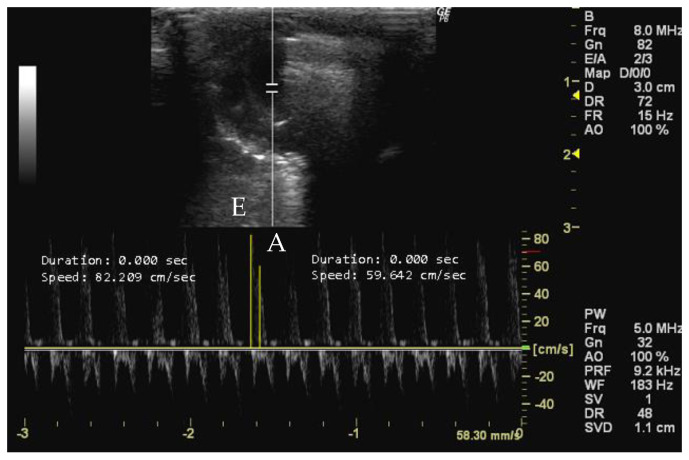
Peak early diastolic transmitral flow (E, cm/s) and peak late diastolic transmitral flow (A, cm/s) measured in 26-week-old female Wistar rats using Doppler mode at early phase diastole, obtained in apical 4-chamber view. The image was obtained using a real-time scanner (Logic P6^®^; General Electric Healthcare, Milwaukee, WI, USA) with a 4–10 MHz linear probe. Measurements were taken using MicroDicom 2023.1 viewer and software.

To measure the peak early and late diastolic transmitral flow velocities (cm/s), it is necessary to obtain an echocardiographic image with a clear view of the mitral valve. The mitral valve is usually visualized between the left atrium and the left ventricle, with the anterior and posterior leaflets clearly visible. The ultrasound cursor must be positioned within the right cardiac chamber of the image, and the velocities of the two peaks are then measured. The larger peak corresponds to the E-wave, and the smaller one represents the A-wave (Figure 9).

The right atrium (RA) and the left atrium (LA) area can be measured in the apical four-chamber view using B-mode during end systole by tracing its inner border and capturing the maximal atrial size. Enlargement is often associated with conditions such as mitral valve disease, atrial fibrillation, or left ventricle dysfunction [89,91]. Similarly, the RA area can be measured in the same apical view using B-mode during end systole by tracing its inner border. Changes in the RA area may indicate conditions like pulmonary hypertension or tricuspid valve disease.

*How to measure RA and LA*

**Figure 10:**
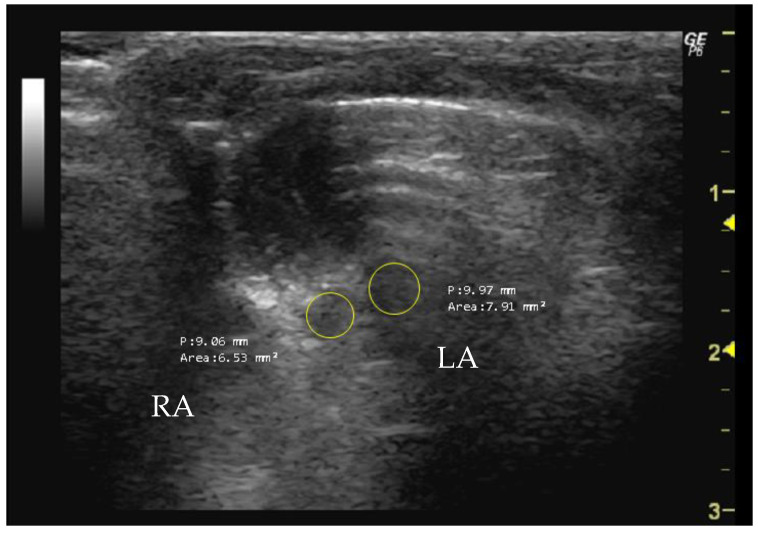
Right atrium (RA, mm^2^) and left atrium (LA, mm^2^) area measured in 26-week-old female Wistar rats using B-mode, obtained in apical 4-chamber view. The image was obtained using a real-time scanner (Logic P6^®^; General Electric Healthcare, Milwaukee, WI, USA) with a 4–10 MHz linear probe. Measurements were taken using MicroDicom 2023.1 viewer and software.

To measure RA and LA (mm^2^), it is necessary to obtain an echocardiographic image that provides a clear visualization of the atria. The RA is located anterior and slightly superior to the RV, while the LA is located posterior and slightly superior to the LV. The cursor should be used to delineate the atrial borders of the RA and LA, thereby allowing the software to calculate the enclosed area (Figure 10).

##### Apical Five-Chamber View

The apical five-chamber view is similar to the four-chamber view but includes the visualization of the aortic root [45]. To simultaneously visualize the LVOT, aortic valve, and aortic root, the transducer should be titled into a shallower angle relative to the chest wall from the apical four-chamber view.

The left ventricular ejection time (LVET, s) is a parameter measurable in this view using Doppler mode. LVET reflects the duration from aortic valve opening to closing during systole [19,45] and indicates left ventricular contraction efficiency [40]. Abnormal LVET suggests various cardiovascular conditions, such as systolic and diastolic dysfunction, aortic valve disorders, or altered preload and afterload conditions [12,40].

*How to measure LVET*

**Figure 11:**
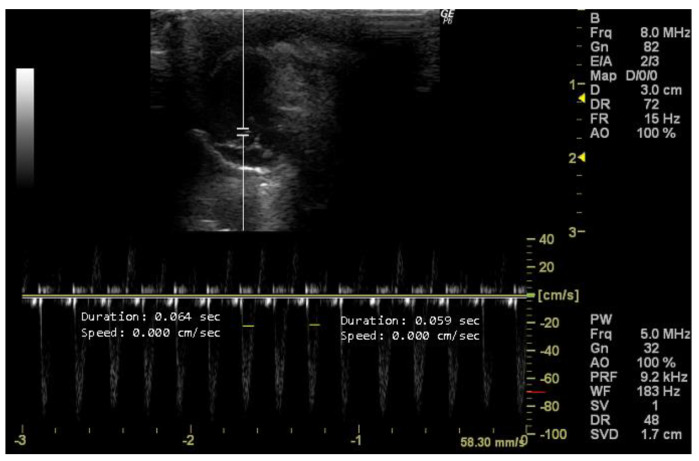
Left ventricle ejection time (LVET, s) duplicate measured in 26-week-old female Wistar rats using Doppler mode, obtained in 5-chamber view. The image was obtained using a real-time scanner (Logic P6^®^; General Electric Healthcare, Milwaukee, WI, USA) with a 4–10 MHz linear probe. Measurements were taken using MicroDicom 2023.1 viewer and software.

To measure LVET, it is necessary to obtain an echocardiographic image that provides a detailed visualization of the LV. The beginning of LV ejection on the Doppler waveform is indicative of the opening of the aortic valve and the onset of systole. The end of LV ejection corresponds to the closure of the aortic valve, marking the end of systole. LVET represents the time integral from the beginning to the end of LV ejection on the Doppler waveform. The program’s cursor is used to measure the wavelength, as illustrated in Figure 11 by the yellow lines.

The aortic velocity–time integral (Ao VTI, cm), also obtained in a 5-chamber view with Doppler mode, is a measurement that provides information about blood flow in the aorta over a complete cardiac cycle [92]. It is the area under the velocity–time curve of blood flow in the aorta during a single cardiac cycle. It represents the total distance traveled by blood per unit of time [65]. The transducer should be placed in the ascending aorta to measure the velocity of blood flow [59].

In sum, these echocardiographic measurements are essential for guiding treatment strategies, monitoring disease progression, and providing valuable insights that inform clinical decision-making. Table 2 summarizes the mode, view, specific measurements, and recommendations pertaining to preclinical rodent models of various diseases or lifestyles where these measurements are pertinent. 

Echocardiography is not only important for the evaluation of heart disease but also for assessing heart function in preclinical models of diseases such as diabetes and cancer [17,93]. Additionally, it contributes to our understanding of the impact of aging on cardiovascular health in preclinical research [94]. Echocardiography plays a crucial role in cancer research by investigating cancer-associated cardiotoxicity induced by anticancer drugs [95]. It enables early detection and monitoring of chemotherapy-induced heart failure [96]. Inflammation and inflammatory diseases also highlight the importance of studying cardiotoxicity [96]. Another example is the study of lifestyle factors, like exercise or obesity, for which monitoring cardiac function is crucial to understanding the heart’s adaptation to physical activity or the pathophysiological changes associated with obesity, respectively [97,98]. Echocardiography is also a crucial tool in studying cardiac complications associated with diabetes in animal models. It allows for early detection and monitoring of diabetic cardiomyopathy [17], providing insights into structural and functional changes in the heart. Echocardiography facilitates the assessment of therapeutic interventions aimed at mitigating cardiovascular risks in diabetes research [99].

**Table 2:** Mode, view, and echocardiographic measurements and their application for diseases/lifestyle models.

Mode	View	Measurement	Disease/Lifestyle Models	Reference
PLAX	B-mode	Aod (mm)	Aging	[100,101]
			Cancer	[102]
			Cardiac diseases	[103]
			Diabetes	[104]
		IVS (mm)	Aging	[101]
			Cardiac diseases	[71,105]
			Cardiotoxicity	[106,107,108,109]
			Diabetes	[104]
			Exercise	[110]
			Obesity	[111]
		LV (mm)	Aging	[101]
			Cardiac diseases	[103,112]
		LVID (mm)	Aging	[101,113]
			Cancer	[102]
			Cardiac diseases	[105,114]
			Cardiotoxicity	[106,107,109,115,116,117]
			Diabetes	[99,118]
			Exercise	[110,119,120,121,122]
			Obesity	[123]
		LVOT (cm)	Aging	[101]
			Cardiac diseases	[71,124]
		LVPW (mm)	Aging	[101,113]
			Cancer	[102]
			Cardiac diseases	[43,103,105,112]
			Cardiotoxicity	[106,107,108,115]
			Diabetes	[104,125]
			Exercise	[110,119,120,122,126]
			Obesity	[111,123,127]
PSAX	B-mode	D1 (mm)	Aging	[128]
			Cardiac diseases	[129]
			Cancer	[130]
		D2 (mm)	Aging	[128]
			Cardiac diseases	[129]
			Cancer	[130]
	M-mode	HR (bpm)	Aging	[113]
			Arthritis	[131]
			Cancer	[102]
			Cardiac diseases	[43,105,114]
			Cardiotoxicity	[106,108,116]
			Diabetes	[104]
			Exercise	[120,121]
			Obesity	[123,127]
	Pulsed Doppler	PA diameter (mm)	Cardiac diseases	[103]
		PA VTI (cm)	Cardiac diseases	[71]
		PAAT (cm/s)	Cardiac diseases	[109,132]
4-chamber	B-mode	LA (mm^2^)	Aging	[100,101]
			Cardiac diseases	[124,133]
			Cardiotoxicity	[109]
		RA (mm^2^)	Aging	[100]
			Cardiac diseases	[124,133]
	M-mode	TAPSE (cm)	Cardiac diseases	[132]
			Cardiotoxicity	[109]
	Pulsed Doppler	A-wave (cm/s)	Aging	[113]
			Cardiac diseases	[43]
			Cardiotoxicity	[109]
			Diabetes	[99,104,125]
			Exercise	[119]
		E-wave (cm/s)	Aging	[113]
			Cardiac diseases	[43]
			Cardiotoxicity	[109]
			Diabetes	[99,104,125]
			Exercise	[119]
5-chamber	Pulsed Doppler	Ao VTI (cm)	Cardiac diseases	[109]
			Diabetes	[104]
		LVET (cm)	Diabetes	[104]

Ao VTI, aortic velocity–time integral; Aod, aorta diameter; D1, left ventricle short-axis diameter parallel; D2, left ventricle short-axis diameter perpendicular; HR, heart rate; IVS, intraventricular septum thickness; LA, left atrium; LV, left ventricle; LV ET, left ventricle ejection time; LVID, left ventricle internal diameter; LVOT, left ventricular outflow tract; LVPW, left ventricle posterior wall thickness; PAAT, pulmonary artery acceleration time; PA, pulmonary artery; PA VTI, pulmonary artery velocity–time integral; PLAX, parasternal long axis; PSAX, parasternal short axis; RA, right atrium; TAPSE, tricuspid annular plane systolic excursion.

### 4.2. Calculable Parameters in Rodent Echocardiography

Echocardiographic parameters are commonly normalized using total body surface area (BSA, cm^2^) as the metric for body size indexation. This accounts for individual differences in body size [134] and provides a more accurate assessment of cardiac dimensions and function compared to absolute measurements [135]. The formula for BSA indexation includes a species-specific constant (k), where k is 9.8 for mice [136], 9.1 for rats [137], and 8.37 for guinea pigs [138]:

$$BSA\;({cm}^{2})=k\times {\left(Body\;Weight\right)}^{2/3}$$

Key calculable parameters in echocardiography include the E/A ratio, which represents blood flow velocities during atrial contraction [139]. The E/A ratio is calculated by dividing the peak E-wave velocity by the peak A-wave velocity. A decreased E/A ratio indicates significantly impaired relaxation, but it may rise again with the progression of diastolic dysfunction as left atrial pressure increases, a phenomenon known as “pseudo-normalization” [140].

$$E/A\;ratio=\frac{Peak\;early\;diastolic\;transmitral\;flow\;(E)}{Peak\;late\;transmitral\;flow\;(A)}$$

The measurement of left ventricle mass (LV mass, mg) is a critical parameter for assessing the size and thickness of the left ventricle of the heart. The left ventricle is responsible for pumping oxygenated blood to the rest of the body, and changes in its mass can indicate various cardiac conditions [64]. This parameter is usually measured using M-mode or 2D echocardiography [141]. LV mass is used to diagnose left ventricular hypertrophy and assess the impact of various medical treatments and interactions on cardiac structure [142]. LV mass can be calculated by using Devereux’s formula corrected for rodents [143]:

$$LV\;mass\;(mg)=1.04\times \left({\left(LVID+LVPW+IVS\right)}^{3}-{\left(LVID\right)}^{3}\right)\times 0.8+0.6$$

where 1.04 is the specific gravity of myocardium, LVID is the left ventricle internal dimension, LVPWd is the left ventricle posterior wall thickness, and IVS is the intraventricular septum thickness. The area–length method is another alternative for determining LV mass. However, while seeming more accurate, it lacks validation for its correlation with 3D-echocardiography-assessed LV weight or mass [144].

Fractional shortening of the left ventricle (FS, %) is a parameter used in echocardiography to assess the contractility and systolic function of the left ventricle of the heart. It is one of several measurements that provide insight into the heart’s ability to pump blood effectively [62]. This parameter is usually measured using M-mode [145]. A normal fractional shortening value typically falls within the range of 25–45% of its diastolic diameter [146]. Lower fractional shortening values suggest reduced systolic function and may be indicative of conditions like heart failure, cardiomyopathy, or myocardial infarction [19]. FS is often used in combination with other echocardiographic measurements, such as ejection fraction (EF), to provide a comprehensive assessment of cardiac function [146]. It is valuable in diagnosing various heart conditions and monitoring changes in ventricular function over time. The formula for calculating FS [12,19,59,62,145] is as follows:

$$FS\;(\%)=\frac{LVIDd-LVIDs}{LVIDd}\times 100$$

where LVIDd is the left ventricle internal dimension in diastole and LVIDs is the left ventricle internal dimension in systole.

LVESV (mL/m^2^) represents the volume of blood in the left ventricle at the end of systole, indicating the minimum volume of blood remaining after contraction. LVEDV (mL/m^2^) is the volume of blood in the left ventricle at the end of diastole, representing the maximum amount of blood the ventricle can hold before contraction [147]. This parameter can be calculated using the following formula [148]:

$$LVESV\;(mL/m^{2})=\left(\frac{7}{2.4+LVIDs}\right)\times LVIDs^{3}$$

$$LVEDV\;(mL/m^{2})=\left(\frac{7}{2.4+LVIDd}\right)\times LVIDd^{3}$$

where LVESV is the left ventricle internal dimension in systole and LVIDd is the left ventricle internal dimension in diastole.

Ejection fraction (EF, %) is obtained by LV measurements in systole and diastole. It is the percentage of blood that the left ventricle ejects into the aorta during systole [12]. EF is important in managing heart failure, as it helps identify individuals who are likely to respond to heart failure medication with lower EF, as well as those who will benefit from device therapy, such as either implanted defibrillators or cardiac resynchronization. EF can be calculated using the following formula:

$$EF\;(\%)=\frac{{\left(LVEDV\right)}^{3}-{\left(LVESV\right)}^{3}}{{\left(LVEDV\right)}^{3}}\times 100$$

Geometric assumption can be a limitation of this technique, but it can be avoided by 3D imaging [26].

Stroke volume (SV, µL) is the amount of blood ejected from the left ventricle of the heart with each systolic cardiac contraction [65]. Some blood that enters the heart near the end of diastole cannot be expelled during systole. The end-systolic volume is the amount of blood that remains in the heart at the end of systole [88]. Stroke volume is influenced by various factors, including fitness levels, sex, heart size, contractility, duration of contraction, preload, and afterload (resistance) [74]. This parameter can be used to evaluate cardiac pump function and organ perfusion [64].

SV (µL)=Aod×AoVTI

where Aod is the aorta diastole and Ao VTI is the aorta velocity–time integral.

By computing SV, it is also possible to calculate cardiac output (CO, mL/min), another essential parameter in echocardiography and cardiology, which measures the volume of blood the heart pumps per unit of time, typically expressed in liters per minute (L/min). It serves as an indicator of heart function and can be used to monitor conditions like heart failure, aortic stenosis, and mitral regurgitation. Normal CO values vary based on factors such as age, body size, and overall health. CO can be calculated using the following formula using the values of stroke volume (SV) and heart rate (HR) [149]:

CO (mL/min)=SV×HR

The eccentricity index is a quantitative measure of the spherical shape during the cardiac cycle, which can be quantified by the ratio of the diameter parallel (D1) and perpendicular (D2) to the septum at diastole [150]. This measurement is particularly relevant in the assessment of cardiac remodeling and function [77] and could be useful in diagnosing variables for patients with suspected pulmonary hypertension [90]. An eccentricity index that significantly differs from one indicates an alteration in the shape of the left ventricle. Increased values may suggest a more elongated or distorted left ventricle, which can be associated with various cardiac conditions [151].

(1) $$Eccentricity\;index=\frac{Left\;ventricle\;diameter\;parallel\;to\;the\;septum}{Left\;ventricle\;diameter\;perpendicular\;to\;the\;septum}$$

Table 3 outlines the calculable echocardiographic parameters and their applicability to disease and lifestyle models. We recommend that both tables (Table 2 and Table 3) be used as reference points by researchers to determine the most appropriate ultrasound parameters for a given preclinical model and disease under study.

## 5. Practical Protocol

In this section, we provide a comprehensive protocol for conducting echocardiographic examinations in studies using rats (Table 4). This protocol delineates the sequential stages involved in the procedure, from the preparation of the animals to the completion of the examination. Each stage is systematically organized to guarantee the accurate and efficient acquisition of cardiac imaging data. Following this protocol ensures the maintenance of consistency and standardization in echocardiographic studies, thereby enhancing the reliability and reproducibility of research findings.

## 6. Applicability of Echocardiography in Preclinical Research

Echocardiography has demonstrated considerable potential as an indispensable tool in preclinical research, particularly in the context of rodent models. The non-invasive nature of echocardiography, coupled with its capacity to provide real-time imaging and a functional assessment of the heart, renders it an optimal technique for a multitude of applications, including disease modeling, assessment of therapeutic efficacy, and toxicology studies.

Echocardiography is a widely employed technique in the development and validation of rodent models of cardiovascular diseases [71]. The visualization and quantification of cardiac structures and functions afforded by this assessment facilitate the study of disease progression and pathophysiology [168]. The evaluation of systolic and diastolic function may be conducted through the measurement of parameters such as EF, FS, and CO [109]. For instance, in rodent models of heart failure induced by methods such as transverse aortic constriction or myocardial infarction, echocardiography is a valuable tool for monitoring disease progression and severity [169]. Similarly, in models of cardiac hypertrophy and cardiomyopathy, as observed in a mouse line that overexpresses the ErbB2 receptor (ErbB2^tg^) in cardiomyocytes, echocardiography provides insights into changes in LV mass, concentric LV hypertrophy, and papillary muscle hypertrophy [170]. Furthermore, echocardiography is employed to examine congenital heart defects in genetically modified rodent models, enabling the visualization of structural abnormalities and the assessment of hemodynamic consequences. This facilitated a deeper comprehension of the development of cardiac anomalies [171].

Echocardiography is also a valuable tool for assessing the efficacy and side effects of novel therapeutic interventions in rodent models [172]. The capacity of echocardiography to furnish quantitative data on cardiac function and structure is pivotal for determining the impact of prospective treatments. As echocardiography is a non-invasive technique, it is regarded as a significant improvement over the invasive methods typically employed to assess cardiovascular function in laboratory animals. It allows for longitudinal assessment of cardiac function, thus enabling the detection of delayed cardiotoxic effects that may not be apparent in short-term studies [172]. For instance, in a rat model of early type 2 diabetes, echocardiography was used to evaluate cardiac structure and function parameters following a diet comprising functional bread enriched with resveratrol. This revealed that maladaptive cardiac remodeling was prevented [104]. Furthermore, echocardiography can also identify changes in cardiac function that may indicate cardiotoxicity, such as a reduced ejection fraction or altered myocardial strain. This is of particular importance in the development of cancer therapeutics, where cardiotoxicity is a common side effect. As an example, the impact of dantrolene (a postsynaptic muscle relaxant) on the cardiotoxicity of doxorubicin in a rat model of breast cancer (female F344 rats with implanted MAT B III breast cancer cells) was assessed using echocardiography. The findings demonstrated that dantrolene reduced doxorubicin-induced alterations in the echocardiographic parameters by 50% [173]. Additionally, this technique is valuable for evaluating the influence of aerobic exercise training on the cardiac remodeling and dysfunction associated with cancer cachexia. This was demonstrated in a study involving CT26 (colon adenocarcinoma cells 26) tumor-bearing Balb/c mice, where aerobic exercise training partially reversed the LV ejection fraction decline [174]. Finally, by monitoring cardiac function at varying doses through echocardiography, researchers can ascertain dose–response relationships and determine the safe therapeutic window for novel compounds, thereby aiding in the determination of the initial human doses [175].

## 7. Conclusions

In sum, echocardiography is a powerful tool in preclinical research for exploring cardiac structure and function non-invasively in rodents. Accurate and reliable data on cardiac morphology, contractility, and hemodynamics can be obtained by considering factors such as anesthesia and positioning to ensure the acquisition. In preclinical research, echocardiography allows the acquisition of valuable pharmacodynamic and safety information that can be translated into clinical practice. Moreover, standardizing echocardiography in preclinical models may also facilitate the early detection of disease characteristics, potentially enhancing diagnosis at earlier stages in humans. This is particularly important as patients are typically diagnosed at later disease stages.

As technology advances, researchers are integrating artificial intelligence, such as Echo2Pheno, to efficiently and accurately analyze conscious mice echocardiograms [176]. This approach promises to save time and resources compared to manual methods of obtaining image-derived phenotypic measurements, but it addresses sampling bias and provides a rapid automated solution. Additionally, the integration of echocardiography with other imaging modalities and omics data could lead to a more comprehensive understanding of cardiac diseases or treatment-related cardiotoxicity. This would allow for the development of more precise therapeutic strategies and accelerate the translation of preclinical findings into clinical applications.

## Figures and Tables

**Table 1:** Advantages and limitations of using echocardiography in the diagnosis and prevention of heart diseases in rats [12,13,14,15,16,19,20,21,22,23,24,25,26,27,28].

Advantages	Limitations
Non-invasive procedure	Time-consuming (more than 20 min)
Portable	Complex and subjective interpretation
Awake or anesthetized animals	Medical specialist training
Real-time imaging	Limited tissue penetration
Very versatile	Low image contrast
Reproducible	Stress factor
Cost-effective	Under anesthesia, heart rate needs to be monitored

**Table 3:** Calculable echocardiographic parameters and their application for diseases/lifestyle models.

Calculable Parameters	Disease/Lifestyle Model	Reference
CO (mL/min)	Aging	[101,109]
	Cancer	[95]
	Cardiac diseases	[109]
	Cardiotoxicity	[116]
	Diabetes	[11]
	Exercise	[110]
	Obesity	[111]
E/A ratio	Arthritis	[119]
	Cardiac diseases	[109]
	Exercise	[106]
	Obesity	[111,123,127]
Eccentricity index	Aging	[128]
	Cardiac diseases	[129]
	Cancer	[130]
EF (%)	Aging	[101,113]
	Arthritis	[152]
	Cancer	[95,102,106]
	Cardiac diseases	[109,112,133,153]
	Cardiotoxicity	[107,108,109,113,115,116,117]
	Diabetes	[43,104,118]
	Exercise	[110,122]
	Obesity	[111,123,127,154]
FS (%)	Aging	[101,113,155]
	Arthritis	[119,152]
	Cancer	[95,102,106]
	Cardia diseases	[109,112,153]
	Cardiotoxicity	[107,108,113,116]
	Diabetes	[43,104,118,125,156]
	Exercise	[110,119,120,122,126,152]
	Obesity	[111,127]
LV mass (mg)	Aging	[109,113]
	Cardiac diseases	[109,112]
	Cardiotoxicity	[117]
	Diabetes	[43,104]
	Obesity	[111,123]
LVEDV (mL/m^2^)	Aging	[113,157]
	Arthritis	[119,158]
	Cancer	[159]
	Cardiac diseases	[112,153,160,161]
	Cardiotoxicity	[113,162]
	Diabetes	[43,163]
	Exercise	[119,164,165]
	Obesity	[166]
LVESV (mL/m^2^)	Aging	[113,157]
	Arthritis	[119,158,167]
	Cancer	[159]
	Cardiac diseases	[112,153,161]
	Cardiotoxicity	[113,162]
	Diabetes	[43,163]
	Exercise	[119,164,165]
	Obesity	[166]
SV (µL)	Aging	[101,109]
	Cardiac diseases	[109,153]
	Cardiotoxicity	[117]
	Diabetes	[43,104]
	Exercise	[110]
	Obesity	[111,123]

CO, cardiac output; EF, ejection fraction; FS, fractional shortening; LV, left ventricle; LVESV, left ventricular end-systolic volume; LVEDV, left ventricular end-diastolic volume; SV, stroke volume.

**Table 4:** Recommended protocol for echocardiographic examination in rats: from animal preparation to echocardiographic procedure and final considerations in the examination process.

Stages	Recommendations
Stage 1: Animal preparation	Turn on all the necessary equipment Record the animal’s weight accurately Administer the anesthesia of preference to the animal in an induction chamber (optional) Ensure continuous sedation by placing a nose cone over the animal (optional) Prepare the animal for the procedure by shaving or applying depilatory cream to the area Position the animal on a heating pad to maintain body temperature
Stage 2: Echocardiographic examination	7. Apply the echo gel to the area and start the examination, adjusting equipment settings as necessary 8. To obtain the PLAX view, position the transducer over the left third of the animals’ chest wall with the notch oriented toward the right shoulder 9. To obtain the PSAX view, rotate the transducer 90 degrees from the PLAX view, aligning the transducer’s notch toward the left shoulder 10. To obtain the apical view, position the transducer at the fifth intercostal space in the left hemithorax. For the four-chamber view, position the transducer at the cardiac apex and direct it toward the right scapula. For the five-chamber view, tilt the transducer into a shallower angle relative to the chest wall from the apical four-chamber view
Stage 3: Final steps in the examination	11. Clean off the echo gel from the animal’s body 12. If applicable, allow the animal to recover from anesthesia on the heating pad; once awake, return the animal to its cage or proceed directly to necropsy 13. Save the collected data and clean and turn off all equipment to conclude the procedure

## Data Availability

No new data were created or analyzed in this study. Data sharing is not applicable to this article.
